# Differentiation of Human Pluripotent Stem Cells into Mesodermal and Ectodermal Derivatives Is Independent of the Type of Isogenic Reprogrammed Somatic Cells

**Published:** 2017

**Authors:** E. S. Philonenko, M. V. Shutova, E. A. Khomyakova, E. M. Vassina, O. S. Lebedeva, S. L. Kiselev, M. A. Lagarkova

**Affiliations:** Vavilov Institute of General Genetics, Russian Academy of Sciences, Gubkina Str. 3, Moscow, 119333 , Russia; Scientific Research Center of Physical-Chemical Medicine, Pirogovskaya Str. 1a, Moscow, 119435, Russia; Kazan State University, Kremlevskaya Str. 18, Kazan, 420008, Russia

**Keywords:** induced pluripotent stem cells, human embryonic stem cells, transcription, hematopoiesis, neurons, methylation

## Abstract

Induced pluripotent stem cells (iPSCs) have the capacity to unlimitedly proliferate and differentiate into all types of somatic cells. This capacity makes them a valuable source of cells for research and clinical use. However, the type of cells to be reprogrammed, the selection of clones, and the various genetic manipulations during reprogramming may have an impact both on the properties of iPSCs and their differentiated derivatives. To assess this influence, we used isogenic lines of iPSCs obtained by reprogramming of three types of somatic cells differentiated from human embryonic stem cells. We showed that technical manipulations *in vitro*, such as cell sorting and selection of clones, did not lead to the bottleneck effect, and that isogenic iPSCs derived from different types of somatic cells did not differ in their ability to differentiate into the hematopoietic and neural directions. Thus, the type of somatic cells used for the generation of fully reprogrammed iPSCs is not important for the practical and scientific application of iPSCs.

## INTRODUCTION

Change in the epigenetic state of a cell using various external conditions fundamentally affects the program of the specialized somatic cell [1, 2]. The most commonly used viral integrative or integration-free methods of reprogramming to a pluripotent state do not substantially affect the genome of the somatic cells subjected to reprogramming [3]. Practical use of induced pluripotent stem cells (iPSCs) for medical or research purposes involves the application of differentiated derivatives of pluripotent cells. The protocols of directed differentiation are aimed primarily at modifying the epigenetic state of pluripotent stem cells (PSCs) by microenvironment conditions mimicking the processes (occurring during) of the individual development of an organism. Thus, the initial epigenetic state and the differences between iPSC lines established even from the same source can have a significant impact on the final result of the differentiation. For example, a total of 25 cell lines was analyzed for the selection of iPSC line-derived retinal pigment epithelium most suitable for transplantation [4], which requires a lot of time and data. In order to study the contribution of the reprogramming process and somatic cell epigenome to the terminal state of iPSCs, as well as optimize the selection of the reprogrammed cell lines, we have developed a system of isogenic lines of pluripotent and somatic cells. The isogenic system of cell lines has allowed us to show that iPSC clones do not leave traces of their tissue-specific origin upon complete functional reprogramming. However, the reprogrammed cells acquired individual epigenetic marks specific to each iPSC clone, indicating that the establishment of pluripotency did not occur in the usual way but through mechanisms different from germline pathways [5]. The appearance of these individual features not related to the reprogramming can be caused by technical manipulations *in vitro *such as cloning, cell sorting, etc.. There is no doubt that a directed influence of such manipulations on the genome can negatively affect further iPSCs application. For example, the possibility of creating banks of reprogrammed cell lines, both personal and immunologically universal lines of iPSCs that would be compatible with a large number of recipients has been widely discussed [6]. However, the question concerning which type of donor cells (skin fibroblasts, blood cells, hair follicle cells, etc.) should be used for reprogramming remains open. According to our results and other studies, isogenic iPSCs derived from different somatic cell types are functionally similar [5, 7]. However, taking into account the fact that they should be further differentiated into specialized types of cells *in vitro*, it is necessary to know how their ability to differentiate would vary.

In the present work, we studied the influence of genetic manipulations, clone selection, and cell sorting *in vitro *on the molecular and genetic properties of iPSCs. In order to do that, we used lines of isogenic somatic cells derived from human embryonic stem cells (hESCs) and their derivatives reprogrammed into iPSCs to compare the ability of isogenic lines of a iPSC line of three different somatic origins to differentiate into the neuronal and hematopoietic directions.

## EXPERIMENTAL

**Cell lines**

We used the cell lines hESM01, hESM01n5 (hereinafter n5), fibroblasts, neurons, retinal pigment epithelial cells differentiated from hESM01n5 (F, N, R, respectively), and the iPSC lines iF, iN, iR obtained by genetic reprogramming of the lines F, N, R, respectively [5].

Human ESC lines HUES 9 [8], H9 [9], iPSC lines endo-iPS12 [10], and IPSRG2L were used in experiments on hematopoietic differentiation [11]. Lines endo-iPSS5 and endo-iPSS9 were obtained by the reprogramming of HUVEC cells using the Sendai virus. The lines of iPSCs were cultured according to [5].

**Media for hematopoietic differentiation**

Medium 1 for embryoid bodies (EB1): Stemline II (Sigma), penicillin-streptomycin (“PanEco”, 5,000 U/ml penicillin and 5,000 U/ml streptomycin), 100 ng/ml VEGF (Prepro Tech), 50 ng/ml BMP-4 (Prepro Tech), and 20 ng/ml FGF (Prepro Tech).

Medium 2 for embryoid bodies (EB2): Stemline II (Sigma), penicillin-streptomycin (“PanEco”, 5,000 U/ml penicillin and 5,000 U/ml streptomycin), 100 ng/ml VEGF (Prepro Tech), 50 ng/ml BMP-4 (Prepro Tech), 20 ng/ml FGF (Prepro Tech), 100x cytokine cocktail CC110 (Stemcell Technologies) or 20 ng/ml SCF (Prepro Tech). Hemangioblasts were cultured in a semi-liquid medium (MHB): Methocult 4436 (Stemcell Technologies), 20 ng/ml FGF (Prepro Tech), 50 ng/ml VEGF (Prepro Tech), 20 ng/ml SCF (Prepro Tech), 20 ng/ml FLT3-L (Prepro Tech), 20 ng/ml TPO (Prepro Tech), 2 μg/ml recombinant HoxB4.

**Hematopoietic differentiation of PSCs**

PSCs cultured in a 35-mm Petri dish (Corning) coated with a Matrigel matrix (BD) were grown to 70% confluence and treated with a 0.05% Trypsin-EDTA solution to a single-cell state. Trypsin was inactivated by the addition of a DMEM medium (“PanEco”) with 10% fetal bovine serum (FBS, Gibco). Embryoid bodies were formed in Aggrewell (Stemcell Technologies) for 24 hours in a mTeSR1 medium (Stemcell Technologies) supplemented with 10 μM Y-27632 (Stemgent). The embryoid bodies were transferred to a 24-well low-adhesion plate (Costar) in a 1-ml volume per well in a EB1 medium and incubated for 48 hours. A 500 μl aliquot was taken from the well, mixed with 500 μl of a EB2 medium and incubated for 48 hours. The embryoid bodies were treated with 0.05% Trypsin-EDTA for 4-6 minutes. Next, trypsin was inactivated by adding the DMEM medium supplemented with 10% FBS. The cells were centrifuged at 200 g for 5 min. The cells (2-5× 10^5^ per volume of not more than 100 μl of a IMDM medium (“PanEco”)) were added to the wells of a 6-well low-adhesion plate (Costar) with a GBS medium using syringes (Stem cell Technologies) with a blunt needle for methylcellulose. The cells were incubated for 6–8 days. Then, additional 2 ml of MHB was added and the cells were incubated for another 2–4 days. For a comparison of the differentiation efficiency, the number of hemangioblast colonies was counted on the 10th day after introduction into MHB.

The ability of hemangioblasts to differentiate into blood cells was tested by the introduction of hemangioblasts into the methylcellulose medium Methocult 4436 (Stemcell Technologies). The result was evaluated after 3 weeks.

**Neuronal differentiation**

Neuronal differentiation into neural progenitors and neurons was performed according to [11]. A FACS analysis of neural progenitors was performed using antibodies to CD56 PE Abcam cat # 2412540 (Sony Biotechnology) (1 : 25 dilution) and isotype control Mouse IgG1PE Abcam cat # 2600560 (1 : 166). For a fluorescence analysis, antibodies against βIII-tubulin in a 1 : 1000 dilution (Abcam) and the secondary antibodies Invitrogen Goat anti Rabbit IgG Alexa Fluor 488 in a 1 : 1000 dilution were used.

Isolation of total RNA from the cell cultures and real-time PCR were performed according to [5].

## RESULTS AND DISCUSSION

**Fig. 1:**
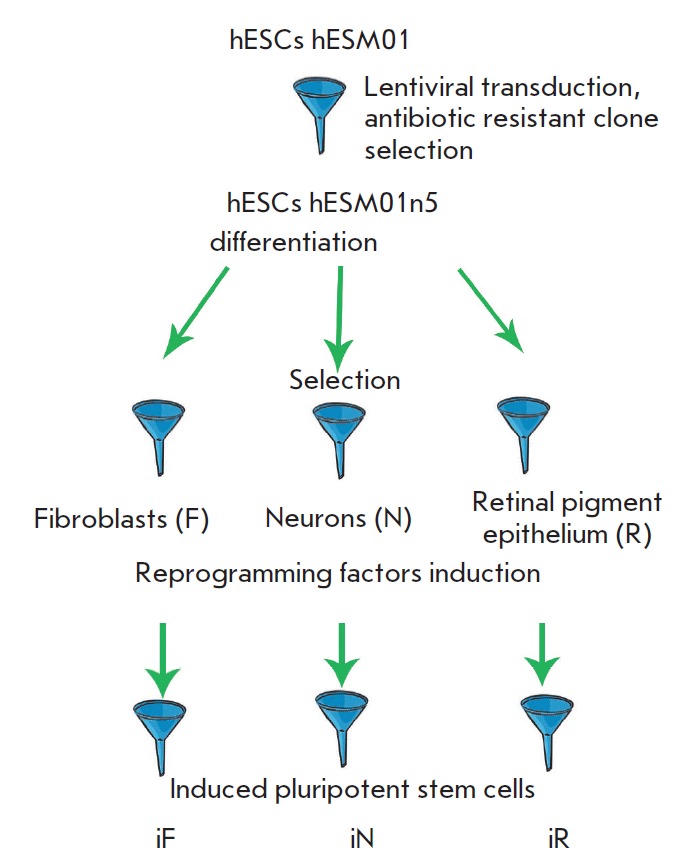
General scheme of the creation of a human isogenic iPSC system depicting critical stages of the passage of cell populations through the bottleneck of selection and cloning.

**Fig. 2:**
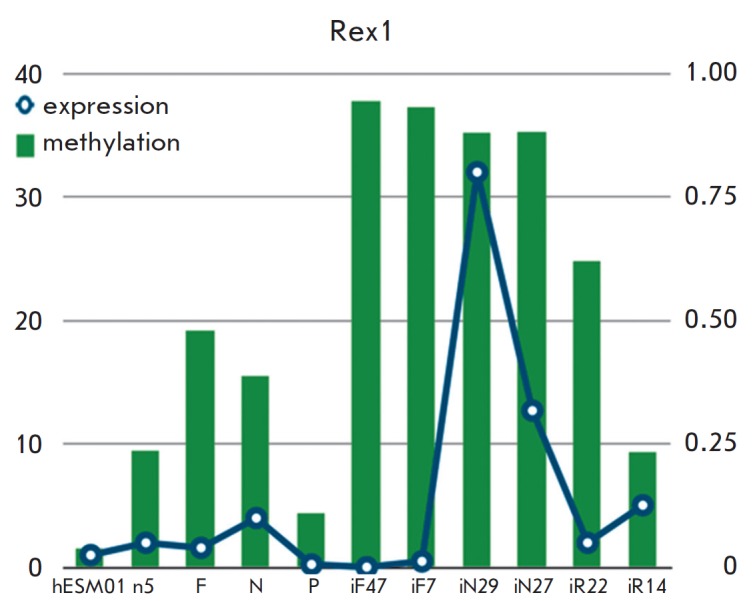
Gradual increase (more than 0.2) of the *Rex1 *methylation level does not correlate with its expression level. Beta-values based on Illumina 450K data were used for the evaluation of the methylation level (*p* < 0.01, fdr < 0.05), while real-time PCR was used to analyze gene expression. (*GAPDH *was used as the reference; the hESM01 line was used to assess the basal level).

Selection and screening of iPSCs do not cause systemic changes in methylation and gene transcription Culturing of human PSCs and, especially, genetic manipulations with these cells are ultimately associated with cell selection and/or cloning. Previously, we established and described an isogenic cell system that allows one to study the molecular mechanisms of reprogramming and differentiation processes [5]. We performed a successive selection of clones and cell sorting several times during the creation of an isogenic system (*Fig. 1*). In relation to this, a question arose as to how manipulations of human PSC cloning in culture can have a systemic impact on gene expression and DNA methylation at the genome-wide level. Expression of certain genes and CpG methylation patterns might have been changed simply because the cells had passed through multiple bottleneck selection procedures. Selective pressure can result in the overexpression of the genes associated with cell survival and confer them an advantage in growth without altering other properties [12]. In the system developed (*Fig. 1*), reprogramming factor genes under the control of an inducible promoter were introduced into the cell line hESM01 and a hESM01n5 clone (hereinafter as n5) was selected which had the lowest level of transgene “leakage” and retained the property of pluropotency [5]. In the next round of the selection, we used magnetic sorting of differentiated n5 derivatives with antibodies to specific markers of the three types of human somatic cells. The last bottleneck happened after the induction of transgenes and selection of iPSC clones (*Fig. 1*). We analyzed the gene expression profile and the level of DNA methylation (database GSE70739) in each of the cell lines obtained at each stage of the system’s establishment. We hypothesized that selective pressure can lead to a successive change in gene expression and/or methylation, providing cells with survival and proliferation advantage. In order to identify the genes and CpG the expression and methylation levels of which gradually increased or decreased during cell selection procedures, the expression/methylation levels in iPSCs were compared between parental lines of somatic cells and isogenic hESC line n5. A gradual increase/decrease of 0.2 for CpG methylation and a 1.5-fold change in the case of gene expression were considered as significant. We analyzed data for 11 cell lines (two hESC lines, three lines of somatic cells, and six lines of iPSCs) the cells of which had been subjected to three bottleneck rounds. We found that transcription of a very small number of genes gradually decreases or increases during cell manipulations, with the transcription level of none of the gene changing in all of the cell lines simultaneously (*Table 1*). This is indicative of the fact that the applied approach, which was identical for all iPSC lines, did not introduce any systematic changes in the cell expression profile, and that the observed expression alterations were accidental. However, the analysis of the methylation profile of isogenic PSC lines and somatic cell lines demonstrated a gradual increase in the methylation level of the *Rex1 *gene (also known as *ZFP42*) (*Table 2*). Currently, there is no definitve information on the function of this gene in early embryonic development. According to some researchers, this gene is considered as a marker of pluripotency [13]. However, mouse ESCs are capable of self-renewal and remain pluripotent even in its absence [14]. As shown earlier, *Rex1 *is expressed in human ESCs even in the case of promoter 50% methylation [15]. Using real-time PCR, we analyzed the *Rex1 *expression level and compared it with the methylation of the promoter region (*Fig. 2*). The expression level of *Rex1*, as well as the level of its promoter methylation, significantly varied in the analyzed pluripotent cell lines. However, we found no correlation between the *Rex1 *methylation level and its expression. For example, the *Rex1 *expression level was more than 3–fold higher in two iN clones (neuron-derived iPSCs) and one iR clone (iPSCs derived from the retinal pigment epithelium) than in all other PSC lines (*Fig. 2*). Thus, the culturing, reprogramming, and selection that led to the hypermethylation of the *Rex1 *promoter region had no impact on gene expression in iPSC lines. This observation additionally confirms the assumption about the auxiliary role of *Rex1 *in maintaining cell pluripotency and also indicates that *Rex1 *expression is not dependent on the methylation status and is extremely heterogeneous in different iPSC lines, as noted previously [12, 15].

**Table 1:** Genes the CpG methylation level of which gradually decreases or increases in the process of hESC differentiation and subsequent reprogramming.

iPSCs	Upregulation	Downregulation
iF	CTGF, TAGLN	SOX15
iN	ACSL4, DDIT4, TIMP1, LOC730278	LFNG
iR	MT1A	

Generally speaking, the obtained results indicate that the procedures of genetic modifications, clone selection, and cell sorting have no systemic impact at the genome-wide level of gene expression and DNA methylation in human PSCs. This conclusion is of serious practical importance due to the possible application of technologies using human PSCs in regenerative medicine.

**Table 2:** Structures of K_V_-channels alone and in complex with charybdotoxin used in homology modeling studies

Increased level of methylation			Decreased level of methylation		
iF	iN	iR	iF	iN	iR
IRX1	ZFP42	AJAP1	AJAP1	MSL3	LOC284661
AJAP1	BANK1	PAX8	CHL1		CD1C
REC8	ZNF454	ZFP42	MARCKS		RTKN
C19orf41	HIST1H1A	SIM1	ZNF311		RAET1L
CBLN4	LOC390595	DPP6	GCM2		GPNMB
ZNF542	ZNF829	GNA14	DPP6		GPNMB
ZFP28	ZNF626	ARHGAP22	TCERG1L		MGMT
LOC390595	ZNF568	FIGNL2	MGMT		MGMT
TMEM132C	ZFP28	TBX5	GALNT9		DNAH9
EBF3		A2BP1	TMEM121		BAHCC1
PTPRN2		CCDC102A	BAHCC1		
ZFP42		HS3ST4	SHISA6		
		ARHGAP23	C22orf34		
		SHISA6			
		TMEM200C			
		MYH14			
		AFF2			

**Comparison of the ability of isogenic PSCs to differentiate into the hematopoietic direction**

**Fig. 3:**
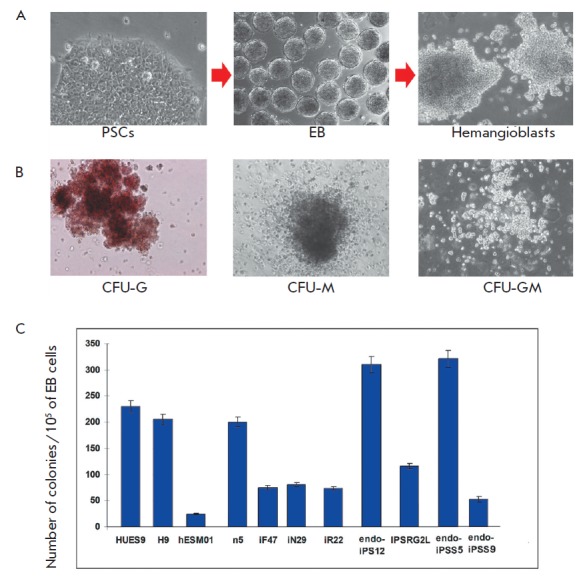
Hematopoietic differentiation of iPSCs. *A *– PSCs, embryoid bodies and hemangioblasts obtained from PSCs. Line n5 is shown. *B *– Examples of hematopoietic colonies formed in methylcellulose. *C *– Comparison of the number of hematopoietic colonies formed in methylcellulose from different PSCs lines on day 10.

The established isogenic system of human PSC reprogramming and differentiation analysis allowed us to prove that one can use somatic cells of any type for reprogramming, since the type of the cells do not make any significant contribution to establishment and maintenance of the pluripotent state [5]. However, taking into account the fact that only differentiated derivatives of PSCs have to be used further, the issue of how the somatic cell type the iPSCs are derived from would affect differentiation efficiency remains open. In order to evaluate this influence, we decided to examine the differentiation efficiency of isogenic human iPSCs derived from fibroblasts (iF), neurons (iN), and retinal pigment epithelium (iR) into hematopoietic cells and neurons. For the evaluation of the effectiveness of hematopoietic differentiation through the stage of embryoid bodies, PSCs were differentiated into early mesodermal derivatives (*Fig. 3A*). The mesodermal derivatives obtained earlier possessed the characteristics of hemangioblasts, since they could differentiate into blood cells (*Fig. 3B*) and endothelium (data not shown). Upon introduction of hemangioblasts into a semi-liquid methylcellulose medium containing hematopoietic cytokines and growth factors, the hemangioblasts formed different types of hematopoietic colonies: erythroid (CFU-E), macrophage (CFU-M), granulocyte (CFU-G), mixed granulocyte-macrophage (CFU-GM), as well as mixed-type colonies CFU-mix, which indicates that hemangioblasts are hematopoietic cells. The examples of such colonies are shown in *Fig. 3B*. The ability of various iPSC lines to differentiate was assessed by the number of hemangioblast colonies formed in MHB on the 10th day. The performed calculation showed that the ability of PSCs to differentiate in the hematopoietic direction greatly varies between the lines but does not depend on their origin. For example, the hESM01 cell line showed the lowest efficiency of hematopoietic differentiation (*Fig. 3B*), while the n5 cell line derived from it was characterized by a significantly higher efficiency of hematopoietic differentiation. The isogenic iPSC lines did not differ in their ability to differentiate despite the fact that they were obtained from different germ layers. Fibroblasts and blood cells belong to the same germ layer. However, the efficiency of iF differentiation into hematopoietic cells was comparable to the isogenic iPSCs of other somatic cell types obtained alongside with them. Other lines that were included into the analysis exhibited different differentiation efficiencies. It should be noted that, in contrast to previously published data, we did not observed a reduced efficiency of human iPSC line differentiation in the hematopoietic direction compared to hESCs [16]. These results indicate that the ability of hematopoietic differentiation is an intrinsic characteristic of each particular PSC line, and that the optimal direction of differentiation can be chosen using, for example, gene cards [17]. The isogenic iPSC lines shared an almost identical ability to differentiate in the hematopoietic direction, since the similarity of the method and simultaneity of reprogramming and culturing, as well as other external conditions, apparently, made a greater contribution to the similarity of the lines than the differences established in their tissue origin.

**Comparison of the ability of isogenic PSCs to differentiate in the neuronal direction**

**Fig. 4:**
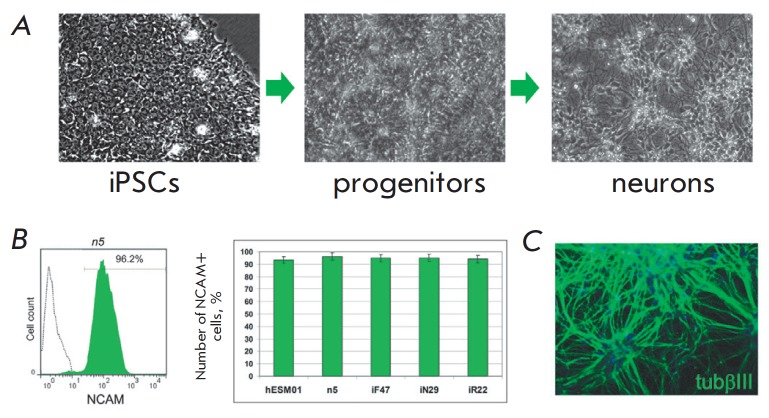
The efficiency of differentiation in the neural direction does not depend on the tissue of origin of isogenic iPSCs. *A *– Scheme of PSC differentiation into neurons. iPSCs differentiate up to the stage of neuronal progenitors and further, in the presence of neurotrophic factors, into neurons. *B *– flow cytometry results of the NCAM+ neuronal progenitors obtained from isogenic hESCs and iPSCs. On the left are the results of flow cytometry of the neuronal progenitors obtained from the n5 line (green): isotype control is not colored. On the right is a summary of the results of flow cytometry of five lines presented as a diagram. *C *– immunohistochemical analysis of neurons differentiated from iPSCs. Green – antibodies to βIII tubulin: nuclei are stained with DAPI (blue)

One of the most popular trends in using differentiated PSC derivatives is the study of the nervous system functioning and therapy of neurodegenerative diseases. In this context, a comparative analysis of the efficiency of the differentiation of isogenic PSCs in the neuronal direction becomes relevant. In order to do this, the original hESC line and iPSCs iN, iF, and iR, which are isogenic to it, were differentiated through the neuronal pathway (*Fig. 4A*). The differentiation efficacy was evaluated by immunocytochemical staining of the cells and flow cytometry at the stage of neuronal progenitors carrying the surface antigen CD56 (NCAM). The developed protocol allowed us to obtain neuronal precursors with high efficiency, with more than 90% of the cells being positive for NCAM (*Fig. 4B*). We found no difference in the differentiation efficiency until the stage of neuronal precursors between PSC lines of different origins. Postmitotic neurons (*Fig. 4C*), which were analyzed by immunohistochemistry for the presence of βIII-tubulin, were obtained during the subsequent differentiation. We also did not find any statistically significant differences between PSC lines in the efficiency of differentiation into postmitotic neurons (data not shown). Thus, as in the case of hematopoietic differentiation, the type of somatic cells used for reprogramming does not play a role in the effective differentiation of PSCs through the neuronal pathway. The obtained results demonstrate a number of important practical conclusions. First of all, the type of somatic cells used for reprogramming is not important in the creation of a bank of allogenic iPSCs for their further application as differentiated derivatives. Blood or skin cells can be obtained from one donor, while neuronal tissue (if available) can be obtained from another donor. Furthermore, fully reprogrammed iPSCs are identical in their differentiation potential. In addition, we have shown that neither genetic manipulations nor selection of PSC clones had a systemic impact on their properties. Undoubtedly, gene expression is more likely associated with changes in chromatin rather than the mutations affecting gene function. This is additionally confirmed by the recently published data on the high genetic stability of PSCs [18]. Despite the fact that there are significant variations in the epigenetic markers of human PSCs that have been identified [19, 20], recent research data suggest that the current methods of human PSCs cultivation allow one to maintain an epigenetic profile over a long period. Thus, PSC lines and their derivatives can already present well-standardized cultures, which opens up the possibility of their practical use.

## Abbreviations

iPSCs: induced pluripotent stem cells
PSCs: pluripotent stem cells
hESCs: human embryonic stem cells
DAPI: 4’,6-diamidino-2-phenylindole
